# Long axial field of view PET/CT in critically ill patients: lessons from a case report

**DOI:** 10.3389/fmed.2023.1347791

**Published:** 2024-01-04

**Authors:** J. H. van Snick, B. van Leer, M. W. N. Nijsten, J. Pillay, R. H. J. A. Slart, A. W. J. M. Glaudemans, N. D. van Rijsewijk

**Affiliations:** ^1^Department of Nuclear Medicine and Molecular Imaging, University Medical Center Groningen, University of Groningen, Groningen, Netherlands; ^2^Department of Critical Care, University Medical Center Groningen, University of Groningen, Groningen, Netherlands; ^3^Groningen Research Institute for Asthma and COPD (GRIAC), University Medical Center Groningen, University of Groningen, Groningen, Netherlands; ^4^Biomedical Photonic Imaging Group, Faculty of Science and Technology, University of Twente, Enschede, Netherlands

**Keywords:** CT, ICU patient, critically ill, imaging procedure, nuclear medicine, PET, FDG

## Abstract

The introduction of new long axial field of view (LAFOV) scanners is a major milestone in positron emission tomography/computed tomography (PET/CT) imaging. With these new systems a revolutionary reduction in scan time can be achieved, concurrently lowering tracer dose. Therefore, PET/CT has come within reach for groups of patients in whom PET/CT previously was undesirable. In this case report we discuss the procedure of a continuous bed motion (CBM) total-body [^18^F]FDG PET/CT scan in an intensive care patient. We emphasize the clinical and technical possibilities with this new camera system, a matched clinical protocol, and the added value of a dedicated team.

## Introduction

The use of ^18^F-fluoro-deoxy-D-glucose ([^18^F]FDG) positron emission tomography computed tomography (PET/CT) in infection and inflammation imaging has increased over the last decade. Inflammatory cells and bacteria involved in infectious and inflammatory processes have a high glycolytic rate and therefore result in [^18^F]FDG accumulation This allows PET/CT to show areas of ongoing infection and inflammation (1, 2).

[^18^F]FDG PET/CT is of added value for a broad range of infectious and inflammatory causes, such as vasculitis, endocarditis, osteomyelitis and especially when the infection or inflammation is of unknown origin (3, 4). The latter patient category suffers from fever and elevated inflammatory markers while the location of the infection or inflammation remains unknown (5). [^18^F]FDG PET/CT imaging allows for the determination of foci of infection and/or inflammation, thereby guiding therapy (6, 7).

Although [^18^F]FDG PET/CT has become an established diagnostic tool in the clinic it is seldomly used within the intensive care setting (8). This is surprising as both infection and inflammation have a high prevalence in this patient population. The two major syndromes defined by infection and inflammation respectively, sepsis and the acute respiratory distress syndrome, comprise 30% of ICU (intensive care unit) admissions (9–11). Furthermore, nosocomial infections occur frequently in critically ill patients contributing to prolonged organ dysfunction and mortality (12–14). In patients with persistent critical illness (i.e., ≥ 10 days of ICU admission) more than 50% develop a new septic episode, frequently with an unknown focus (15–18). The fact that PET/CT is not widely used in ICU patients is due to the perceived complexity of patients, the comprehensive patient preparation and extensive scanning time (8, 19).

Recent advances in PET/CT technology, such as ultra-high sensitivity mode in long axial field of view (LAFOV) PET/CT systems, have led to a dramatic reduction in acquisition time and administered activity (20–23). These technological improvements remove a perceived barrier to more routine use of PET/CT in ICU patients.

In this case report, we demonstrate the use of the new LAFOV PET/CT system in an ICU patient. The technical and clinical aspects and benefits will be discussed.

## Case

A 67-year-old female was admitted to the ICU due to a Hemophilus influenza sepsis complicated by a heart tamponade, caused by a pericarditis, resulting in an in-hospital cardiac arrest. Successful resuscitation was achieved by pericardiocentesis and placement of pericardial drains. An arthroscopy with rinsing was performed for a bacterial arthritis of the right knee, from which Hemophilus influenza was cultured. Antibiotic therapy with ceftriaxone was started. Subsequently a contrast enhanced CT scan of the thorax was performed showing a bilateral pleural effusion. After an initial phase of recovery, the patient relapsed with an inflammatory profile including fever and a CRP of 128 mg/L, despite continued antibiotic treatment. To identify possible secondary foci, a request for [^18^F]FDG PET/CT examination was made with emphasis on the upper airway since all problems had started with a painful throat. Previously no imaging focusing on this area had been performed.

In the nuclear medicine department, a dedicated physician assistant (PA) is available for ICU patients to manage logistics and patient preparation. The patient was prepared following international standards, including 4 h fasting period and discontinuation of glucose infusion (24, 25).

The patient was scheduled with priority the next day on a LAFOV (106 cm) Biograph Vision Quadra PET/CT scanner (Siemens Healthineers, Knoxville, TN, United States). A total of 145 MBq (2 MBq/kg) of [^18^F]FDG was intravenously administered at the ICU department after checking the blood glucose (6.4 mmol/L). After 30 min, the patient was transferred to nuclear medicine department according to our in-house protocol for transfer of critically ill patients. For transfer from the bed to the scanning table, a hoist mechanism is used. During the scan, the patient was fully sedated, required mechanical ventilation and vasopressor support through multiple intravenous lines. The tubing for the intravenous lines was extended to 2.5 m (26). Prior to the start of the scan, a scan-table movement test was conducted to confirm the placement and unhindered movement of the patient with the lines and tube. Once these checks were confirmed, the PET/CT scan was started 60 min post injection (27).

In order to take full advantage of the new features of the Quadra system, the scan was performed in 12 min continuous bed mode (CBM) and in ultra-high sensitivity (UHS) mode (23). Continuous bed mode facilitates the slow and gentle movement of the scan bed through the gantry, permitting 195 cm to be scanned in one pass without limitation of the 106 cm FOV. In this case, it was crucial to focus not only on the primary area of concern (the throat), but also on the known foci in the knee that required examination. The data were reconstructed with our clinical standard protocol: Ordinary-Poisson Ordered-Subsets Expectation–Maximization and TrueX+TOF (UltraHD-PET) 4 iterations, 5 subsets, matrix 440 × 440 voxel 1.65 × 1.65 mm, no filter, and Ultra High Sensitivity. Images were reviewed by a nuclear medicine specialist in Syngo.Via VB60 using MM-oncology (Siemens Healthineers, Erlangen, Germany) (28).

**Figure 1:**
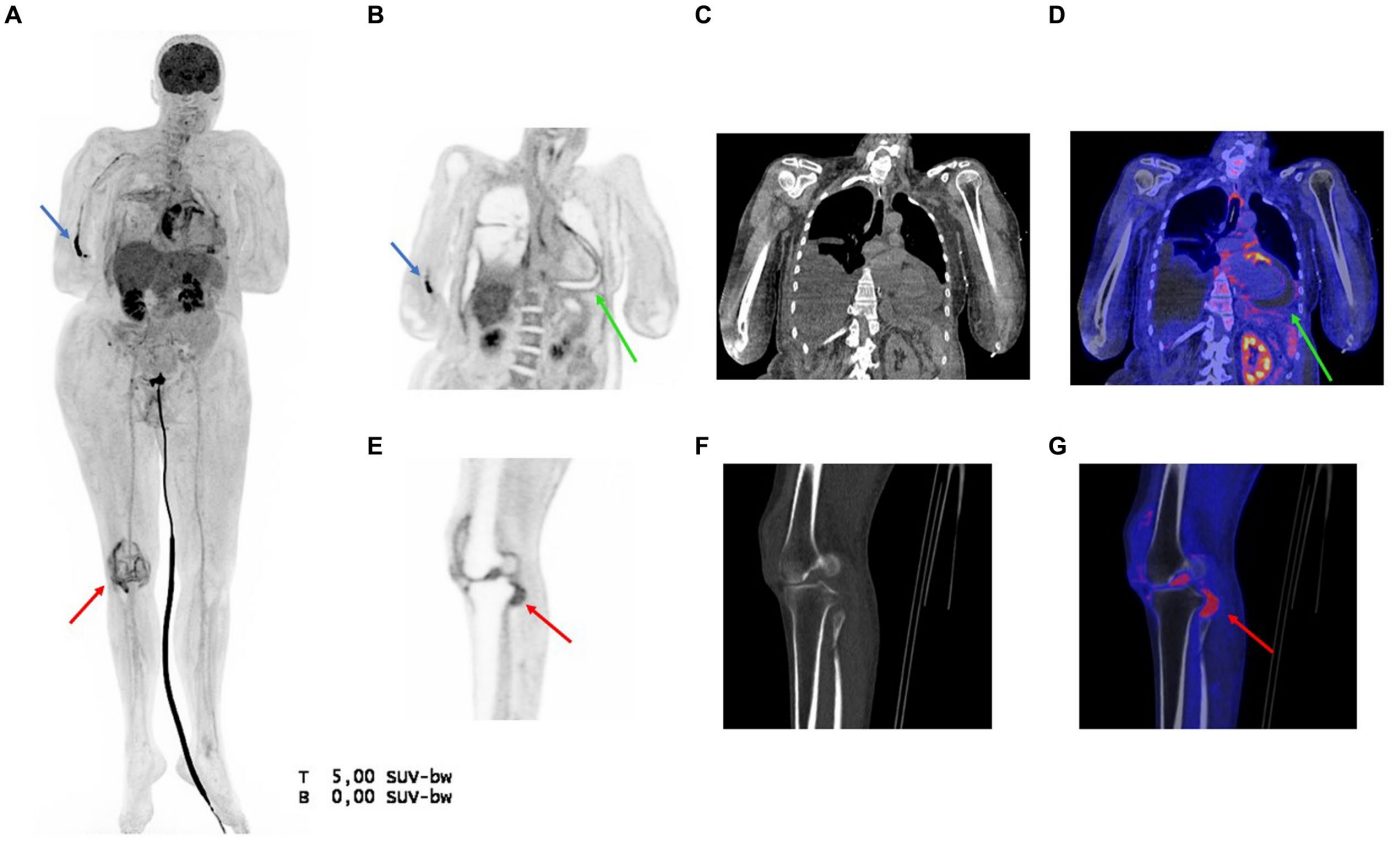
**(A)** Maximum Intensity Projection (MIP) showing pathological [^18^F]FDG uptake in the pericardial cavity and right knee (red arrow) and [^18^F]FDG enhancement in the cephalic vein, possibly caused by a thrombophlebitis (blue arrow). Furthermore, physiological uptake can be seen in the liver, kidneys, and urinary catheter tract. **(B–D)** Coronal PET, low-dose CT, and PET/CT fusion image of the thorax, respectively, showing clear pericardial effusion (green arrow) with slightly elevated [^18^F]FDG uptake in the pericardial layers. The pleural effusion showed no tracer uptake. **(E–G)** Sagittal PET, low-dose CT, and PET/CT fusion image of the right knee (red arrow), respectively, showing increased [^18^F]FDG uptake in the synovial space due to the Hemophilus Influenza infection.

The [^18^F]FDG PET/CT did not show an infectious focus in the head and neck area besides some reactive lymph nodes. Furthermore, persisting pericardial fluid without [^18^F]FDG uptake was seen despite continuous drainage (Figures 1B–D). [^18^F]FDG enhancement was seen in the pericardial layers, mainly around the right atrium, most likely due to some remaining pericarditis (Figures 1A,B,D). Bilateral pleural effusion did not show [^18^F]FDG uptake, which was in line with negative cultures of this fluid obtained directly after the PET/CT. However, some increased uptake in the pleural sheets could indicate pleuritis. [^18^F]FDG uptake was seen in the right cephalic vein indicating a possible thrombophlebitis (Figure 1A). In the synovial space of the right knee clear [^18^F]FDG enhancement was visible, indicating the earlier diagnosed synovitis (Figures 1A,E,G).

Concurrently with the PET/CT scan, blood cultures were taken, later showing *Enterococcus faecium*. The central line was removed shortly before the PET/CT, which later showed to be the source, and vancomycin therapy was started. The [^18^F]FDG uptake pattern was classified as a reactive remainder of the initial infection. Together with the negative pleural punction and the central line infection as a cause for the deterioration, ceftriaxone and vancomycin treatment was stopped. The patient rapidly recovered.

## Discussion

This case illustrates the challenges of performing a PET/CT scan in ICU patients due to the complexity of mechanical ventilation and vasopressor support required by the patient, presence of intravenous lines and the necessary planning, logistics and transport. The added value of performing a [^18^F]FDG PET/CT scan in ICU patients was demonstrated. Thanks to the PET/CT scan another localization of the *Hemophilus influenza* could be excluded facilitating safe tapering of antibiotic treatment. However, more importantly, we showed that, with the recent advancement in technology, a PET/CT can be performed relatively easily in ICU patients using a LAFOV PET/CT camera, which is mainly achieved by reduction of the scan time (up to 2 min for one bed position) (29).

Another advantage of the newest function of the LAFOV PET/CT system is the added value of scanning in CBM mode, allowing to scan a total body while the table moves gently and remains comfortable for the patient, with also less risk for lines and tubes to get stuck or tangled compared to scanning total body using the multiple bed positions method. This bed position method is used in older type of scanners, statically scanning one bed position before moving to the next, resulting in relatively sudden movements between positions. Besides, it needs large overlap in bed positions for sufficient fusion between the low dose CT and PET scan, demanding more acquisition time.

In this case, a total acquisition time of 12 min was used since this was the first time an ICU patient was scanned using the LAFOV PET/CT system in CBM mode. By further adapting the procedure, a total of 6–7 min acquisition time seems to be feasible in this patient group, without compromising image quality and diagnostic value. In comparison, the acquisition time would have been approximately 30 min on a conventional PET/CT system. Furthermore, due to the limited sensitivity of conventional PET/CT’s, a higher radioactivity dose must be administered with these types of camera systems. This results not only in a higher radiation dose for the patient but also for the ICU staff (30, 31). A dose reduction as low as 1 MBq/kg for a LAFOV PET/CT has already been suggested (20).

The successful execution of an [^18^F]FDG PET/CT procedure for critically ill patients requires a specialized team with sufficient experience and expertise. Key members of this team include an ICU physician, an ICU nurse and competent PET/CT technologists. Furthermore, it is crucial to have a dedicated facilitator to ensure smooth communication and coordination between the two departments. This role is fulfilled in our institution using a physician assistant of the nuclear medicine department. Furthermore, a standard operating procedure should be available for both departments.

In conclusion, the utilization of LAFOV PET/CT yields a nearly similar duration for transport and scanning when compared with an ordinary CT scan. Therefore, using this system, PET/CT has become more accessible for use in critically ill patients. However, before implementation, perceived barriers in scanning complex ICU patients need to be addressed by research into safety, patient preparation, and logistics. Eventually, this may result in international practice guidelines.

## Data availability statement

The datasets presented in this article are not readily available because the raw data is protected by privacy laws. Fully anonymized data is available on reasonable request. Requests to access the datasets should be directed to the corresponding author.

## Ethics statement

The requirement of ethical approval was waived by Medical ethical committee UMCG for the studies involving humans. The studies were conducted in accordance with the local legislation and institutional requirements. The participants provided their written informed consent to participate in this study. Written informed consent was obtained from the individual(s) for the publication of any potentially identifiable images or data included in this article.

## Author contributions

JS: Conceptualization, Methodology, Writing – original draft. BL: Conceptualization, Supervision, Writing – review & editing. MN: Writing – review & editing. JP: Writing – review & editing. RS: Writing – review & editing. AG: Supervision, Writing – review & editing. NR: Conceptualization, Supervision, Writing – review & editing.
